# A dose-response study of the association between MRI-derived liver proton density fat fraction and prevalent T2DM in postmenopausal women with abnormal glucose metabolism

**DOI:** 10.3389/fendo.2026.1777573

**Published:** 2026-05-21

**Authors:** Yong-li Zheng, Wei Zhang, Tao Yuan, Lei Gao, Xiao-na Zhu, Ying Liu, Rui-qing Shi, Gui-fen Han, Guan-min Quan

**Affiliations:** 1 Department of Medical Imaging, The Second Hospital of Hebei Medical University, Shijiazhuang, Hebei, China; 2 Department of Medical Imaging, Hebei Medical University Third Hospital, Shijiazhuang, Hebei, China

**Keywords:** alanine aminotransferase, aspartate aminotransferase, prediabetes, proton density fat fraction, triglyceride-glucose index, type 2 diabetes mellitus

## Abstract

**Objective:**

This study aimed to investigate the dose-response association between magnetic resonance imaging-derived hepatic proton density fat fraction (MRI-derived PDFF) and the type 2 diabetes mellitus (T2DM) prevalence in postmenopausal women with abnormal glucose metabolism, evaluate its utility in risk stratification, while examine the potential mediating role of liver enzymes and to explore the nonlinear relationship between MRI-derived PDFF and insulin resistance assessed by the Triglyceride-Glucose (TyG) index, in this specific population.

**Methods:**

In this cross-sectional study, 96 postmenopausal women with abnormal glucose metabolism (46 with prediabetes, 50 with T2DM) were included. Hepatic and pancreatic fat fractions were measured by MRI, and visceral fat mass (VFM) was quantified by quantitative computed tomography. Inter-observer agreement was assessed using intraclass correlation coefficients. Multivariable logistic regression, with sequential adjustment for age, BMI, total cholesterol, and VFM, was used to assess the association between liver fat fraction and T2DM, treating hepatic proton density fat fraction of the left lobe (FF-LL) as both a continuous variable and in quartiles. The discriminative ability of FF-LL was evaluated by ROC analysis. Mediation analysis explored the potential role of liver enzymes, and restricted cubic splines examined the relationship between FF-LL and the TyG index.

**Results:**

In postmenopausal women with abnormal glucose metabolism, those with T2DM demonstrated a more unfavorable metabolic profile compared to those with prediabetes. Inter-observer agreement was excellent for liver-related parameters and VFM (ICCs: 0.977–0.984), but moderate for pancreatic fat fraction (ICC range: 0.602–0.637). FF-LL was significantly higher in the T2DM group compared to the prediabetes group. ROC analysis demonstrated that FF-LL had good discriminative ability for T2DM (AUC = 0.816). The optimal cutoff value was 6.3%, yielding a sensitivity of 80.0% and a specificity of 87.0%. As a continuous variable, each unit increase in FF-LL was associated with a 15% to 20% higher prevalence of T2DM across adjusted models. A significant dose-response trend was observed across increasing quartiles of FF-LL. In addition, mediation analysis showed that both alanine aminotransferase (ALT) and the AST (aspartate aminotransferase)/ALT ratio partially mediated the association of FF-LL with T2DM in postmenopausal women, accounting for 22.0% and 24.9% of the total effect, respectively (both P = 0.040). Additionally, restricted cubic spline analysis revealed a significant nonlinear association between FF-LL and the TyG index (overall P < 0.001, P for nonlinearity = 0.007), characterized by a threshold effect with a steep increase in FF-LL when TyG exceeded approximately 8.75.

**Conclusion:**

In postmenopausal women with abnormal glucose metabolism, FF-LL is an independent risk factor for T2DM, exhibiting a dose–response relationship partially mediated by liver enzymes. With an optimal cutoff of 6.3%, FF-LL demonstrates good discriminative ability and may serve as an effective biomarker for T2DM risk stratification. Additionally, a significant nonlinear association is observed between FF-LL and the TyG index in this population.

## Introduction

Type 2 diabetes mellitus (T2DM) represents a major global public health challenge, with its rising prevalence placing a significant strain on healthcare systems worldwide (1). In this context, precise risk stratification within high-risk populations is critical for implementing targeted prevention strategies to delay disease onset (2). Postmenopausal women represent one such high-risk group requiring particular attention. Due to a marked decline in estrogen levels, they experience a centripetal shift in body fat distribution and reduced insulin sensitivity, leading to a significantly elevated risk of T2DM (3). Among this population, prediabetes serves as a critical intermediate stage in the transition from normal glucose metabolism to overt diabetes, offering a valuable “window of opportunity” for intervention. Effective management of this stage is of decisive significance in halting disease progression (4). Therefore, focusing on postmenopausal women with abnormal glucose metabolism (including both prediabetes and diabetes) to investigate their specific risk biomarkers holds important clinical value (5). However, traditional T2DM risk prediction tools, such as clinical questionnaire-based risk scores or conventional biochemical indicators, although of some value, have limitations in reflecting individual-level pathophysiological states (e.g., organ-specific fat deposition), and their predictive efficacy and specificity warrant improvement (6). Consequently, the exploration of more accurate, objective, and convenient novel biomarkers has become a focal point of current research.

Substantial evidence underscores the strong association between metabolic dysfunction-associated Steatotic liver disease (MASLD) and T2DM, as both conditions share insulin resistance as a core pathophysiological mechanism and often co-occur (7). As the central organ of energy metabolism, the liver, when affected by excessive fat deposition, not only exhibits the hallmark of MASLD but also acts as a key driver of systemic insulin resistance and glucose metabolic dysregulation (8, 9). Therefore, precise quantification of liver fat content in postmenopausal women with abnormal glucose metabolism represents a promising critical biomarker indicating underlying diabetes risk, facilitating more accurate risk stratification (10).

While liver biopsy remains the histological gold standard for diagnosing hepatic steatosis, its invasiveness, sampling variability, and high cost limit its widespread clinical application (11). Conventional noninvasive tools, including ultrasound and serum-based indices, demonstrate limited sensitivity in detecting mild steatosis. In response to this need, MRI-derived proton density fat fraction (MRI-PDFF) has emerged as the reference standard for noninvasive quantification of liver fat, demonstrating high precision, accuracy, and reproducibility (12, 13). Although the overall association between hepatic steatosis and dysglycemia is well established (14), the dose-response relationship between hepatic PDFF and T2DM risk remains incompletely understood, especially in postmenopausal women with abnormal glucose metabolism. Elucidating this relationship is a critical prerequisite for translating hepatic PDFF into a valid biomarker for clinical risk stratification. Furthermore, the specific pathophysiological pathways through which liver fat accumulation underlies the progression of diabetes in this population have not yet been fully elucidated. Specifically, the induction of hepatocellular damage (manifested by elevated circulating liver enzymes) and the exacerbation of systemic insulin resistance are considered two key potential mechanisms (15, 16). In order to provide mechanistic insight into how hepatic fat accumulation may influence T2DM risk, it is worth noting that the triglyceride glucose (TyG) index, as a reliable and convenient alternative indicator of insulin resistance, holds significant utility in large-scale epidemiological studies (17). Previous studies have indicated that the TyG index exhibits a significant stepwise increase with the severity of hepatic steatosis, suggesting a complex, potentially nonlinear relationship between these variables that warrants further investigation (18).

Therefore, this study aims to establish a precise dose-response relationship between hepatic steatosis quantified by MRI-PDFF and the prevalence of T2DM in a cross-sectional cohort of postmenopausal women with abnormal glucose metabolism.

In addition, we seek to evaluate the potential mediating role of liver enzymes and to explore the nonlinear relationship between liver fat fraction and insulin resistance in this specific population. Elucidating these relationships has the potential to facilitate precise risk stratification within this cohort and to offer a robust evidence base for implementing early interventions for high-risk individuals.

## Materials and methods

### Study design and participants

This study was conducted from April 2024 to November 2025 and was approved by the Ethics Committee. The inclusion criteria were as follows: female patients aged 50–80 years who underwent health examinations at our institution and required both liver PDFF assessment and biochemical testing. The exclusion criteria were as follows: acute liver injury, drug-induced chronic liver disease, pre-menopausal female patients, severe cardiovascular disease, obesity secondary to other causes, hepatic or renal dysfunction, autoimmune diseases, active infectious diseases, history of malignant tumors, history of paralysis, as well as current smokers or individuals consuming alcohol.

The diagnosis of prediabetes and diabetes was based on the 2021 American Diabetes Association criteria. Prediabetes was defined as a hemoglobin A1c (HbA1c) level of 5.7–6.4% (19). After applying the detailed inclusion and exclusion criteria, 96 patients were enrolled in the final study cohort, comprising 50 diagnosed with T2DM and 46 with prediabetes.

### mDIXON-quant examinations and measurement

The proton density fat fraction (PDFF) was quantified using a 3.0-T MRI system (Ingenia; Philips Healthcare) with a three-dimensional multi echo chemical shift-encoded sequence (mDIXON-Quant; Philips). For both the liver and pancreas, axial images were acquired during a single breath-hold.

The sequence parameters for each organ are detailed below. For liver imaging, a breath-hold acquisition was employed with the following technical parameters: repetition time (TR) was set to the shortest possible value; echo time (TE) was 1.15 ms; echo spacing was set to the shortest possible value; the field of view (FOV) was 400 × 267 × 325 mm; the acquisition matrix was 156 × 122; the voxel size was 2.5 × 1.5 × 5.0 mm with a slice thickness of 5 mm; the flip angle was 3°; the number of signal averages (NSA) was 1; and the total acquisition time was 14 seconds. For pancreatic imaging, a similar breath-hold technique was used with the following parameters: repetition time (TR) was set to the shortest possible value; echo time (TE) was 6 ms; echo spacing was set to the shortest possible value; the field of view (FOV) was 375 × 132 × 120 mm; the acquisition matrix was 156 × 122; the voxel size was 2.5 × 2.5 × 3.0 mm with a slice thickness of 3 mm; the flip angle was 3°; the number of signal averages (NSA) was 1; and the total acquisition time was 14 seconds.

**Figure 1:**
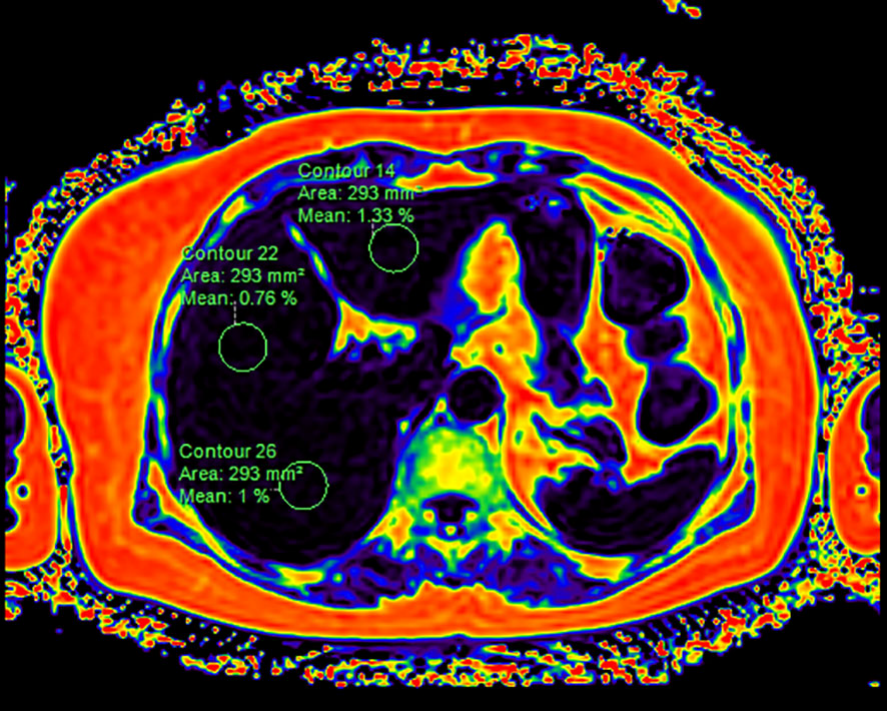
Schematic diagram illustrating the placement of regions of interest (ROIs) for hepatic proton density fat fraction (PDFF) measurement.

All regions of interest (ROIs) were manually delineated on the FF maps. On the axial slice of the liver showing the largest cross-sectional area, circular ROIs of approximately 300 mm² each were manually placed within the left hepatic lobe, the right anterior hepatic lobe, and the right posterior hepatic lobe. Care was taken to avoid including bile ducts, blood vessels, and the liver margins (**Figure 1**). Similarly, for the pancreas, circular ROIs were manually placed on the axial slice showing the largest cross-sectional area at four anatomical locations: the head, the body, and the tail and uncinate process. To ensure accurate quantification, these ROIs were carefully delineated to encompass as much pancreatic parenchyma as possible while consistently avoiding pancreatic vasculature and organ margins. The mean FF of the entire liver and pancreas was subsequently calculated based on the values obtained from these multiple ROIs. Hepatic and pancreatic fat fractions were quantified by two blinded independent observers, with no access to participant data.

### QCT examinations and image analysis

QCT scans and image analysis were performed for all participants. Abdominal CT scans were acquired using an asynchronous quantitative CT protocol on a 128-slice multidetector CT scanner (Somatom Sensation, Siemens, Erlangen, Germany). Participants were positioned supine, with their arms raised above their heads. The scanning range extended from the inferior margin of the 12th thoracic vertebra to the superior margin of the 4th lumbar vertebra. Details of the acquisition parameters are as follows: tube voltage 120 kV, tube current 125 mAs, pixel size 0.78 mm, pitch 0.8, matrix 512 × 512, field of view 500 mm, and slice thickness 1 mm. The images were transferred to a dedicated QCT workstation and analyzed using the body composition analysis module (Mindways Software Inc., Austin, TX, USA; Version 5.10). Visceral fat mass at the level of the L3 vertebral body(L3-VFM) was measured semi-automatically with manual adjustments where necessary. This measurement was based on reconstructed images with a slice thickness of 1 mm. Visceral fat mass was quantified by two independent observers blinded to the participants’ clinical characteristics and diagnostic status, ensuring objectivity of the measurements.

### Clinical data collection and laboratory examinations

Following an overnight fast of at least 8 hours, venous blood samples were collected from the antecubital vein of all participants the next morning for biochemical analysis. The measured parameters included alanine aminotransferase (ALT), aspartate aminotransferase (AST), AST/ALT ratio, alkaline phosphatase (ALP), total cholesterol (TC), triglycerides (TG, mmol/L), low-density lipoprotein cholesterol (LDL-C), very low-density lipoprotein cholesterol (VLDL-C), fasting plasma glucose (FPG, mmol/L), urea, creatinine (Cr), uric acid (UA), calcium (Ca), phosphorus (P), magnesium (Mg), and glycated hemoglobin (HbA1c). In addition, general clinical information, including age, sex, and body mass index (BMI), was collected. Following biochemical analysis, the TyG index was calculated using the following formula: TyG = ln [Fasting Triglycerides (mg/dL) × Fasting Plasma Glucose (mg/dL)/2].

### Statistical analysis

R software (version 4.2.2), along with MSTATA software (www.mstata.com) were used for statistical analysis. GraphPad Prism (v10.1.2, GraphPad Software, San Diego, California, USA, www.graphpad.com) was used for drawing figures. Normality of continuous variables was assessed using the Shapiro-Wilk test. Data are presented as mean ± standard deviation (SD) or median (interquartile range, IQR), for normally and non-normally distributed variables, respectively. Group comparisons were performed using independent samples t-tests for parametric data and Mann-Whitney U tests for non-parametric data, as appropriate. P < 0.05 was considered statistically significant.

### Inter-observer agreement

Inter-observer agreement for hepatic fat fraction, pancreatic fat fraction, and visceral fat mass assessed by two independent blinded observers was assessed using the intraclass correlation coefficient (ICC) and Bland-Altman plots, where an ICC value greater than 0.75 was considered indicative of good reproducibility.

### Multivariable logistic regression

The association between FF-LL levels and T2DM was assessed using multivariable logistic regression. FF-LL was analyzed in two ways: first as a continuous variable, and second categorized into quartiles (Q1–Q4, with Q1 as the reference group) to evaluate a potential dose-response relationship. Covariate selection was guided by prior literature and biological plausibility. To isolate the independent contribution of liver fat deposition to diabetes prevalence in this high-risk population, a sequential adjustment approach was employed. To progressively isolate the unique contribution of liver fat deposition to diabetes prevalence in this high-risk population, a sequential adjustment strategy was employed. Model 1 included age and BMI to account for the physiological decline in β-cell function and insulin sensitivity with advancing age, as well as the confounding effect of overall obesity. Model 2 further adjusted for TC to control for potential confounding by dyslipidemia—a common comorbidity strongly associated with insulin resistance. In Model 3, visceral fat mass was additionally incorporated to evaluate whether the association between liver fat and T2DM was independent of visceral adiposity, given that BMI does not accurately reflect fat distribution. This stepwise adjustment approach was designed to progressively isolate the independent contribution of liver fat deposition.

### ROC analysis

To further investigate the utility of FF-LL as a risk stratification biomarker for T2DM in postmenopausal women, we evaluated its discriminative ability by calculating the area under the receiver operating characteristic curve (AUC).

### Mediation analysis

To investigate the potential mediating role of liver enzymes, a mediation analysis was performed within the association between FF-LL and T2DM. In this analysis, FF-LL was treated as the exposure, T2DM as the outcome, and liver enzyme levels as the putative mediators. This analysis quantified both the direct effect of FF-LL on T2DM and its indirect effect mediated through liver enzymes.

### Restricted cubic splines

To further explore the potential nonlinear relationship between liver fat and insulin resistance, the association between FF-LL and the TyG index was assessed using restricted cubic splines.

## Results

### Baseline and clinical characteristics

After screening according to the inclusion and exclusion criteria, a total of 96 postmenopausal women were finally enrolled, including 46 patients with prediabetes and 50 patients with T2DM. The baseline characteristics of the patients were summarized in **Table 1**. Overall, compared to the prediabetes group, patients with T2DM demonstrated a more unfavorable metabolic profile, exhibiting higher levels of lipid parameters (TC, HDL-C, LDL-C), liver injury markers (AST/ALT), as well as increased fasting blood glucose, FPG, HbA1c and TyG index.

**Table 1:** Patient demographics and baseline characteristics.

Characteristic	Overall N = 96	Prediabetes N = 46	T2DM N = 50	p-value
age(year)	63 (59, 69)	62 (56, 67)	63 (59, 69)	0.094
Height(cm)	160.0 (158.0, 165.0)	162.0 (160.0, 166.0)	160.0 (157.0, 163.0)	0.015*
Weight(kg)	63 (59, 67)	64 (58, 69)	63 (60, 67)	0.757
BMI(kg/m²)	24.55 (22.60, 26.40)	23.85 (22.20, 26.60)	24.70 (23.10, 26.20)	0.415
ALT(U/L)	19 (15, 26)	19 (14, 22)	20 (15, 29)	0.089
AST(U/L)	20.0 (17.0, 23.6)	20.0 (18.0, 23.0)	19.0 (17.0, 24.0)	0.869
AST/ALT	1.06 (0.84, 1.21)	1.16 (0.95, 1.41)	0.98 (0.83, 1.18)	0.016*
ALP(U/L)	72 (57, 81)	76 (61, 83)	70 (56, 78)	0.291
TC(mmol/L)	5.03 ± 0.96	5.45 ± 0.91	4.65 ± 0.85	<0.001*
TG(mmol/L)	1.22 (0.86, 1.79)	1.21 (0.89, 1.63)	1.22 (0.85, 2.00)	0.315
HDL-C(mmol/L)	1.47 ± 0.30	1.58 ± 0.28	1.38 ± 0.28	<0.001*
LDL-C(mmol/L)	2.81 (2.34, 3.23)	3.06 (2.61, 3.48)	2.54 (2.10, 3.02)	<0.001*
VLDL(mmol/L)	0.55 (0.39, 0.81)	0.55 (0.40, 0.74)	0.55 (0.39, 0.91)	0.317
Urea(mmol/L)	5.01 (4.39, 5.74)	4.71 (4.33, 5.65)	5.19 (4.53, 6.06)	0.153
Cr(μmol/L)	61 (55, 70)	60 (55, 68)	61 (55, 70)	0.477
UA(μmol/L)	287 (252, 334)	303 (253, 348)	283 (249, 327)	0.218
Ca(mmol/L)	2.35 (2.28, 2.40)	2.35 (2.31, 2.39)	2.33 (2.27, 2.40)	0.342
P(mmol/L)	1.21 ± 0.13	1.20 ± 0.11	1.21 ± 0.15	0.704
Mg(mmol/L)	0.90 (0.84, 0.93)	0.91 (0.88, 0.93)	0.88 (0.83, 0.94)	0.214
FPG(mmol/L)	6.32 (5.60, 8.20)	5.62 (5.32, 6.17)	8.17 (6.56, 9.67)	<0.001*
HbA1c	6.20 (5.90, 7.00)	5.90 (5.80, 6.10)	6.95 (6.30, 7.80)	<0.001*
TyG	8.78 (8.38, 9.26)	8.66 (8.34, 8.82)	9.19 (8.57, 9.59)	<0.001*
FF(LL)%	3.9 (2.4, 7.0)	2.7 (2.2, 5.0)	5.4 (2.8, 8.0)	0.015*
FF(RAL)%	3.8 (2.6, 8.2)	3.6 (2.7, 6.3)	3.9 (2.3, 8.7)	0.681
FF(RPL)%	3.0 (1.8, 6.7)	2.8 (1.8, 4.8)	3.1 (1.8, 7.8)	0.557
PDFF(L)%	3.4 (2.2, 7.1)	3.2 (2.2, 5.2)	4.1 (2.2, 8.7)	0.263
FF(UP)%	4.7 (2.4, 9.5)	5.2 (3.0, 10.2)	4.0 (2.2, 9.4)	0.221
FF(PH)%	3.6 (1.9, 6.3)	3.3 (1.3, 6.9)	3.7 (2.6, 5.4)	0.413
FF(PB)%	4.0 (2.2, 6.5)	4.5 (2.6, 7.8)	3.6 (2.1, 5.3)	0.078
FF(PT)%	3.9 (2.3, 7.5)	5.0 (2.1, 7.6)	3.7 (2.3, 6.7)	0.590
P(FF)%	4.57 (2.79, 6.79)	5.34 (3.09, 7.27)	3.95 (2.73, 6.48)	0.250
L3-VFM (g)	13.2 ± 5.0	13.8 ± 5.3	12.8 ± 4.7	0.328

Data presented as mean±standard deviation or median (inter-quartile range). ALT, Alanine Aminotransferase, AST, Aspartate Aminotransferase, AST/ALT, AST to ALT Ratio, ALP, Alkaline Phosphatase, TC, Total Cholesterol, TG, Triglycerides, HDL-C, High-Density Lipoprotein Cholesterol, LDL-C, Low-Density Lipoprotein Cholesterol, VLDL, Very Low-Density Lipoprotein, Urea, Urea, Cr, Creatinine, UA, Uric Acid, Ca, Calcium, P, Phosphorus, Mg, Magnesium, FPG, Fasting Plasma Glucose, HbA1c, Glycated Hemoglobin A1c, TyG, Triglyceride-Glucose Index, FF(LL), PDFF of the Left Hepatic Lobe, FF(RAL), PDFF of the Right Anterior Hepatic Section , FF(RPL), PDFF of the Right Posterior Hepatic Section, PDFF(L), PDFF of the Whole Liver, FF(PH), PDFF of the Pancreatic Head, FF(PB), PDFF of the Pancreatic Body, FF(UP), PDFF of the Uncinate Process, PT(FF), PDFF of the Pancreatic Tail, P(FF), Mean Pancreatic Fat Fraction, L3-VFM(g), Visceral Fat Mass at the L3 Vertebral Level,Statistically significant (P* < 0.05).

**Figure 2:**
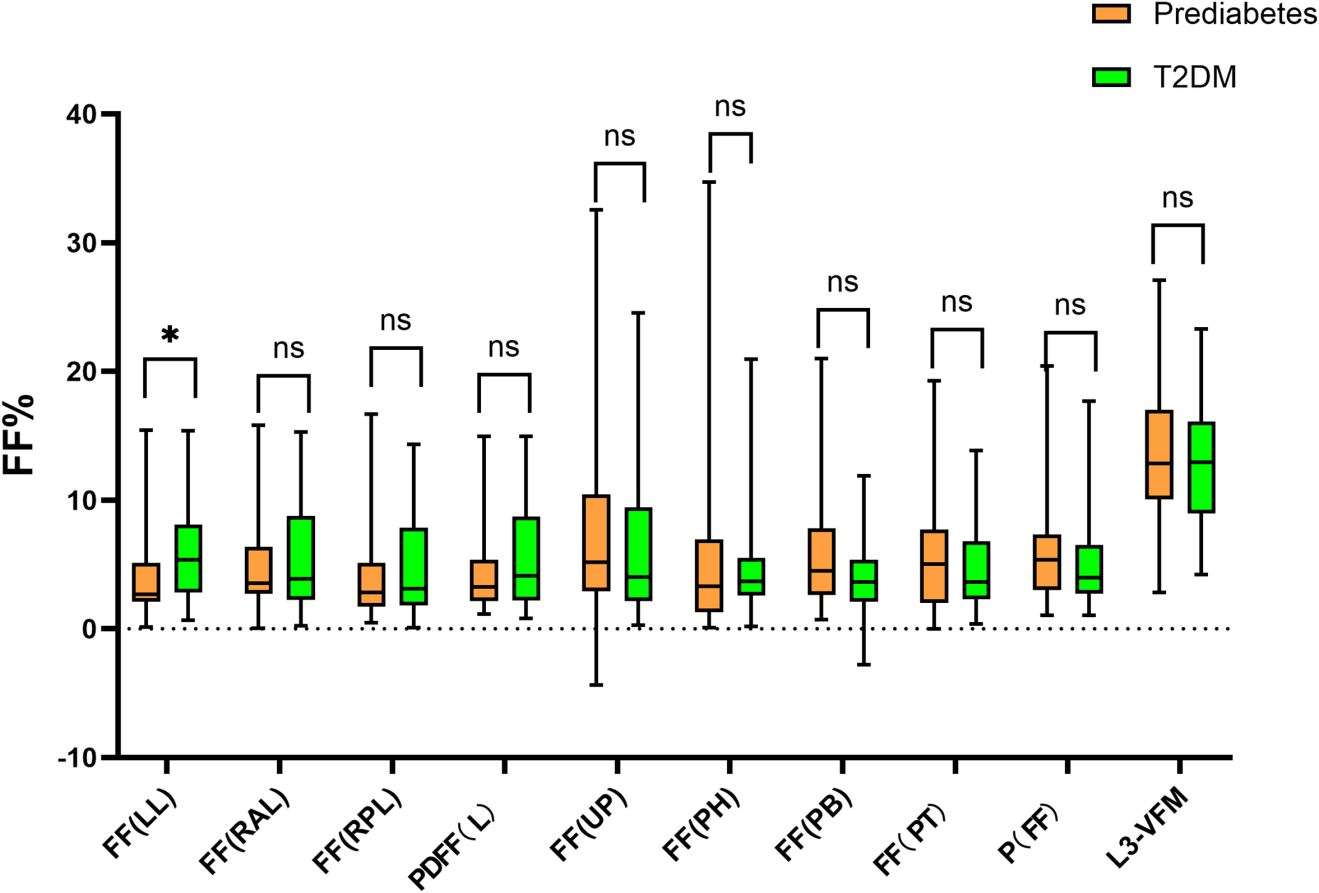
Distribution of hepatic PDFF, pancreatic PDFF, and visceral adipose tissue mass (box plots). FF(LL), PDFF of the Left Hepatic Lobe; FF(RAL), PDFF of the Right Anterior Hepatic Section; FF(RPL), PDFF of the Right Posterior Hepatic Section; PDFF(L), PDFF of the Whole Liver; FF(PH), Fat Fraction of the Pancreatic Head; FF(PB), Fat Fraction of the Pancreatic Body; FF(UP), Fat Fraction of the Uncinate Process; PT(FF), PDFF of the Pancreatic Tail; P(FF), Mean Pancreatic Fat Fraction; L3-VFM (g), Visceral Fat Mass at the L3 Vertebral Level. * indicates P < 0.05, meaning the result is statistically significant.

Regarding the hepatic fat fraction (FF), which was the focus of this study, patients with T2DM showed a significantly higher FF in the left hepatic lobe (FF-LL) compared to the prediabetes group [5.4 (2.8, 8.0) vs. 2.7 (2.2, 5.0); P = 0.015]. The FF in the right anterior lobe, right posterior lobe, and the whole liver also appeared higher in the T2DM group, although these differences did not reach statistical significance. There were no significant differences in the pancreatic fat fraction or L3-VFM between the two groups (**Figure 2**).

### Inter-observer agreement assessment

**Figure 3:**
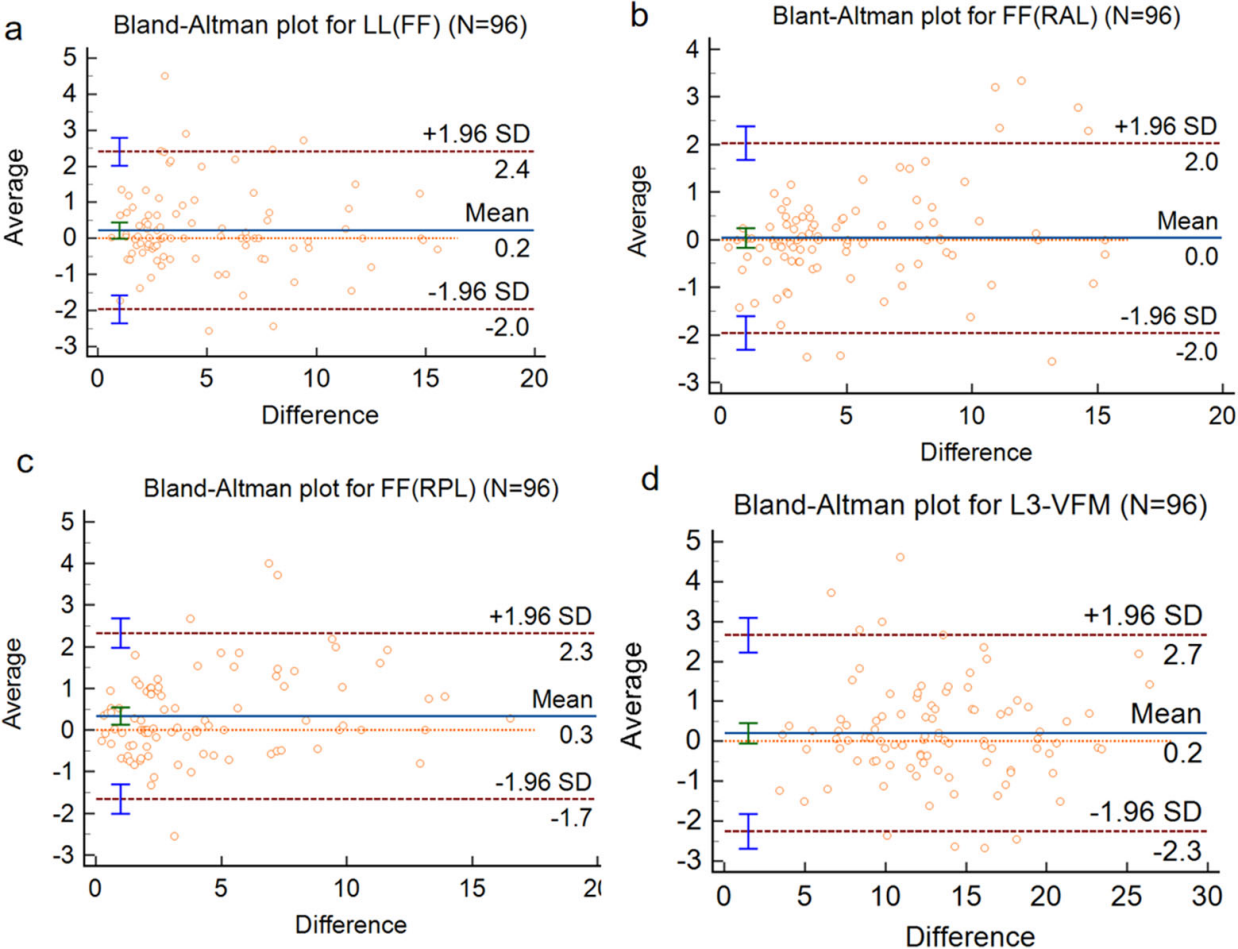
Bland-Altman plot for inter-observer agreement of **(a)** FF(LL); **(b)** FF(RAL); **(c)** FF(RPL) and **(d)** L3-VFM.

Excellent agreement was exhibited across all liver measurements and VFM. Specifically, Intraclass Correlation Coefficient (ICC) values indicated excellent reliability: 0.977 (95% CI: 0.965–0.985) for FF-LL, 0.983 (95% CI: 0.974–0.988) for fat fraction of the Right Anterior Lobe, 0.981 (95% CI: 0.971–0.987) for fat fraction of the Right Posterior Lobe, and 0.984 (95% CI: 0.976–0.990) for VFM. Bland-Altman plots revealed that differences between observers were distributed symmetrically around the mean difference, with no systematic bias (**Figure 3**). In contrast, pancreatic measurements showed moderate agreement, with ICC values ranging from 0.602 to 0.637. Collectively, these findings demonstrate that liver measurements and VFM are highly reproducible and reliable across independent observers.

### Association between left lobe hepatic fat fraction and T2DM risk

Logistic regression analyses showed that LL-FF is an independent risk factor for T2DM in postmenopausal women with abnormal glucose metabolism, and its effect remains stable after adjusting for age, BMI, TC, and VFM. As shown in **Table 2**, when analyzed as a continuous variable, FF-LL was significantly associated with higher prevalence of T2DM across all adjusted models. In the adjusted model with additional adjustment for age and BMI (Model 1), each unit increase in FF-LL was associated with a 15% higher prevalence of T2DM (OR = 1.15, 95% CI: 1.02–1.31, P = 0.025). This association strengthened with further adjustment for TC in Model 2 (OR = 1.20, 95% CI: 1.05–1.38, P = 0.01) and remained significant after additional adjustment for L3-VFM in Model 3 (OR = 1.18, 95% CI: 1.03–1.34, P = 0.013). This indicates that after adjusting for different sets of covariates, each unit increase in FF-LL was associated with a 15% to 20% increase in the prevalence of T2DM.

**Table 2:** Multivariable logistic regression analysis of the association between FF-LL and T2DM.

Characteristic	Model 1			Model 2			Model 3		
	OR	95% CI	p-value	OR	95% CI	p-value	OR	95% CI	p-value
FF(LL) (continuous)	1.15	1.02, 1.31	0.025*	1.20	1.05, 1.38	0.010*	1.18	1.03, 1.34	0.013*
FF(LL)									
Q1	—	—		—	—		—	—	
Q2	0.84	0.26, 2.70	0.765	0.96	0.27, 3.35	0.949	0.88	0.28, 2.80	0.833
Q3	1.12	0.35, 3.56	0.849	1.16	0.33, 4.03	0.815	1.32	0.42, 4.20	0.638
Q4	2.56	0.76, 8.65	0.130	3.43	0.89, 13.18	0.072	3.18	0.94, 10.70	0.062
P for trend			0.117			0.077			0.049*

FF-LL was analyzed both as a continuous variable and in quartiles (Q1-Q4, with Q1 as the reference). Model 1: adjusted for age and BMI; Model 2: further adjusted for TC; Model 3: further adjusted for L3-VFM. CI, confidence interval; OR, odds ratio.Statistically significant (P* < 0.05).

When FF-LL was categorized into quartiles, compared with the lowest quartile (Q1), the highest quartile (Q4) showed a progressively increased risk of T2DM, with an OR of 3.18 (95% CI: 0.94–10.70, P = 0.062) in the fully adjusted Model 3. Although the Q4 estimate did not reach conventional statistical significance, a significant dose–response trend was observed across quartiles in Model 3 (P for trend = 0.049), indicating that higher liver fat content is associated with a stepwise increase in T2DM risk, independent of age, overall obesity, dyslipidemia, and visceral adiposity. These findings demonstrate a clear dose–response relationship between FF-LL quartiles and T2DM prevalence, independent of established confounders.

### Discriminative ability of FF-LL for T2DM

**Figure 4:**
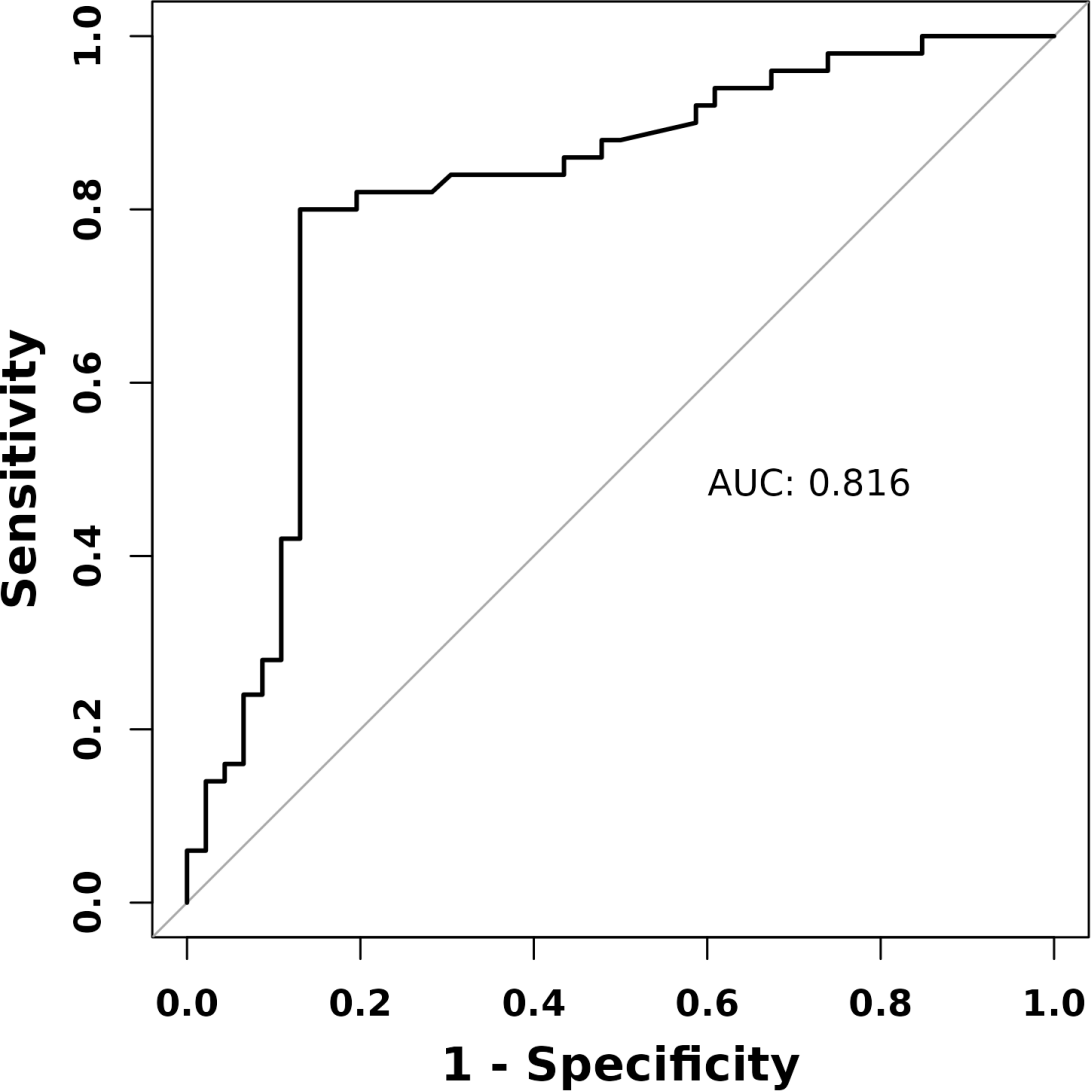
Receiver operating characteristic (ROC) curve of FF-LL for distinguishing T2DM from prediabetes in postmenopausal women.

ROC curve analysis demonstrated that FF-LL showed strong discriminative performance for T2DM risk stratification, with an AUC of 0.816 (**Figure 4**). The optimal cutoff value was established at 6.3%, corresponding to a sensitivity of 80.0% and a specificity of 87.0%. These results demonstrate that FF-LL can effectively distinguish T2DM from prediabetes in postmenopausal women with abnormal glucose metabolism.

### Mediation analysis of liver enzymes

**Figure 5:**
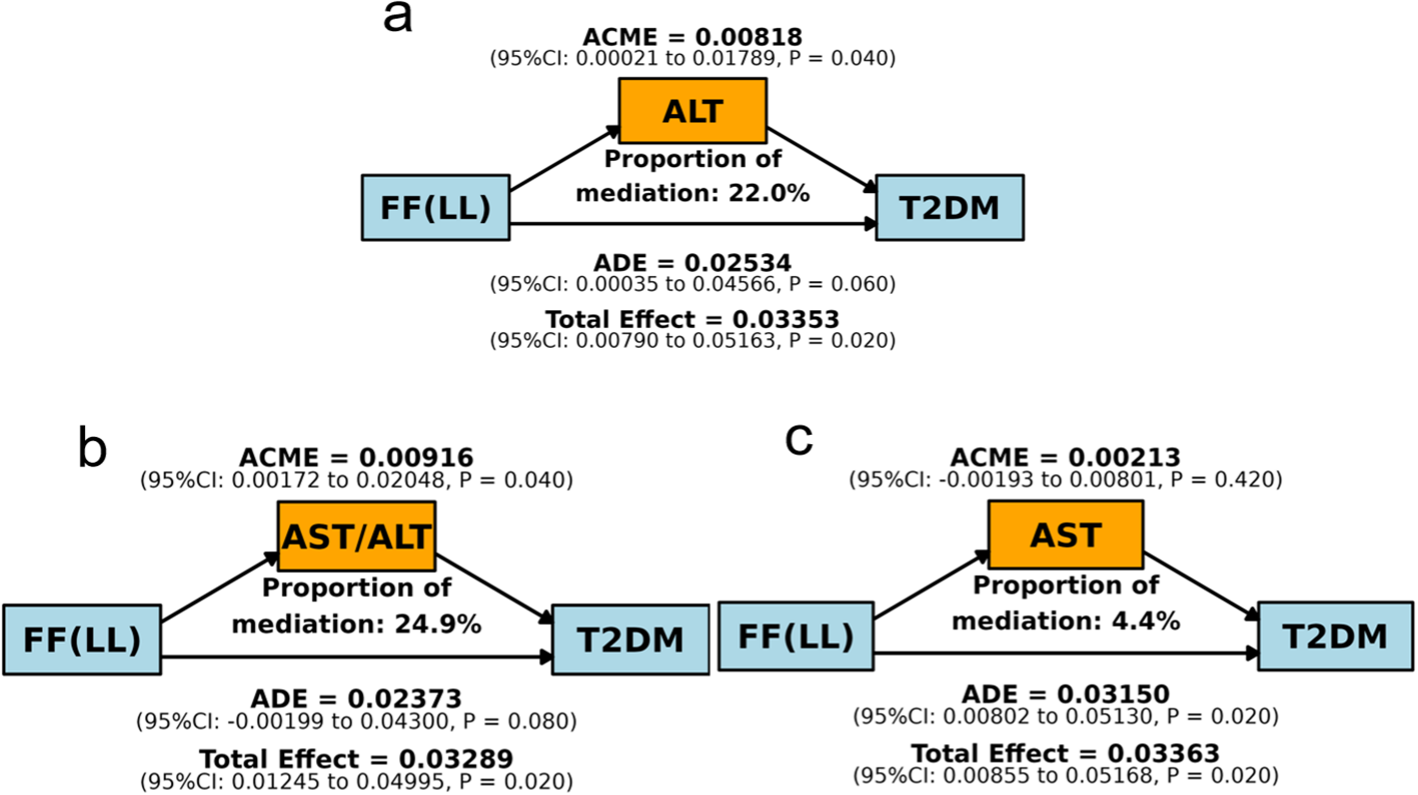
Mediation analysis illustrating the role of ALT **(a)**, AST/ALT **(b)** and AST **(c)** in the association between FF-LL and T2DM.

Mediation analysis was conducted to examine the role of liver enzymes in the association between FF-LL and T2DM. The analysis was adjusted for age, BMI, TC, and L3-VFM, and a significant indirect effect was observed for ALT (coefficient = 0.01, P = 0.04), which accounted for 22.0% of the total association between FF-LL and T2DM (**Figure 5A**). Similarly, the AST/ALT ratio also showed a significant mediating effect (coefficient = 0.01, P = 0.04), which accounted for 24.9% of the total association between FF-LL and T2DM (**Figure 5B**). In contrast, AST did not demonstrate a significant indirect effect (P = 0.42; **Figure 5C**). In summary, the pathway from hepatic steatosis to T2DM was partially mediated by markers of liver injury, specifically ALT and the AST/ALT ratio.

### Nonlinear association between insulin resistance and FF-LL

**Figure 6:**
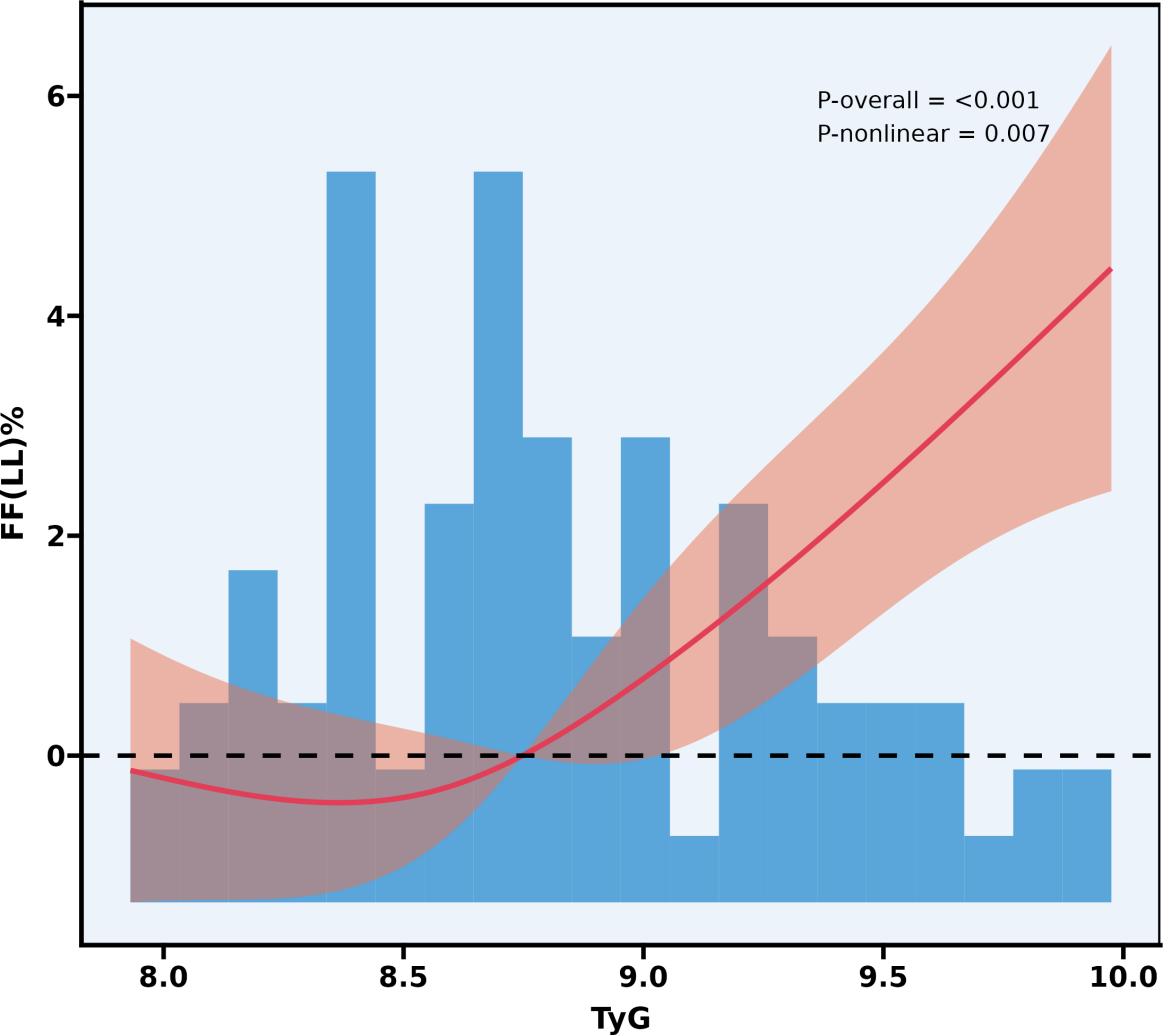
Restricted cubic spline analysis of the association between TyG index and FF-LL.

Restricted cubic spline analysis revealed a significant nonlinear association between the TyG index and FF-LL (overall P < 0.001, P for nonlinearity = 0.007) (**Figure 6**). FF-LL did not change linearly with increasing TyG, but instead exhibited a distinct nonlinear pattern: in the TyG range of 8.0 to 8.75, FF-LL showed a mild initial decrease followed by a gradual recovery, with an overall small magnitude of change. Beyond this inflection point, the curve rose steeply, and the 95% confidence interval remained consistently above the 0 reference line, indicating a statistically significant positive association between high TyG levels and elevated left hepatic lobe fat fraction. The histogram in the figure illustrates the distribution of TyG index values in the study population.

## Discussion

The increasing incidence of both MASLD and T2DM in postmenopausal women highlights a pressing need for more refined and standardized risk stratification tools. MASLD, characterized by hepatic steatosis, shares insulin resistance as a common pathophysiological link with T2DM (20). In this context, FF-LL serves as a quantitative imaging biomarker for hepatic steatosis—the core component of MASLD —and is therefore of particular value. Given this pathophysiological connection, investigating the association between FF-LL and T2DM may help elucidate whether liver fat quantification can contribute to diabetes risk stratification. To address this gap, the present study evaluated this association in postmenopausal women with abnormal glucose metabolism. The results showed that FF-LL was significantly higher in the T2DM group than in the prediabetes group. A significant dose-response relationship was observed: for every 1% increase in FF-LL analyzed as a continuous variable, there was an associated 15%–20% higher risk of T2DM. ROC analysis identified an optimal cutoff value of 6.3% for FF-LL in diagnosing T2DM, with an AUC of 0.816, a sensitivity of 80.0%, and a specificity of 87.0%. Mediation analysis revealed that ALT and the AST/ALT partially mediated the association between FF-LL and T2DM, accounting for 22.0% and 24.9% of the total effect, respectively. Furthermore, a significant nonlinear relationship characterized by a threshold effect was observed between FF-LL and the TyG index, wherein FF-LL exhibited a sharp increase once the TyG index exceeded approximately 8.75.

Our baseline analysis revealed that postmenopausal women with T2DM present a more adverse metabolic profile compared to women with prediabetes. Specifically, this profile is characterized by elevated lipid parameters, glycemic markers and increased insulin resistance. This finding corroborates previous studies (21). It can be attributed to the interplay between postmenopausal estrogen deficiency and the core pathophysiological mechanisms of T2DM. Estrogen loss exacerbates visceral adiposity, dyslipidemia, and insulin resistance. Meanwhile, the insulin resistance and beta-cell dysfunction inherent to T2DM further impair glycemic control and lipid metabolism. Together, these factors contribute to a more adverse metabolic profile, consistent with the established pathophysiological mechanisms underlying T2DM.

In postmenopausal women with T2DM, FF-LL was significantly higher than that in the prediabetes group, highlighting the critical role of ectopic hepatic fat deposition in the progression from prediabetes to overt diabetes. Although the right lobe and whole-liver PDFF also showed increasing trends in the T2DM group, these differences did not reach statistical significance. This pattern suggests spatial heterogeneity of hepatic fat deposition, which is consistent with findings reported by Zhao et al. (22). Several factors may account for the superior sensitivity of FF-LL in detecting intergroup differences. First, from an imaging perspective, the left lobe is less affected by cardiac motion and breathing artifacts than the right lobe, particularly in older postmenopausal women, leading to more reliable and reproducible measurements. Second, from a pathophysiological standpoint, the left lobe receives a greater proportion of portal venous blood rich in dietary lipids and gut-derived factors, which may render it more susceptible to diet-induced fat accumulation and insulin resistance. In contrast, the right lobe, being larger and more influenced by systemic metabolic signals, may exhibit a more attenuated response to early metabolic deterioration (23, 24). Collectively, these findings support the use of FF-LL as a sensitive imaging biomarker for assessing diabetes risk in postmenopausal women with abnormal glucose metabolism.

Logistic regression analyses provided robust evidence for a positive, dose-response relationship between FF-LL and the prevalence of T2DM. This association remained significant across progressively adjusted models that accounted for age, BMI, TC, and VFM. The persistence of this relationship suggests that the impact of hepatic steatosis on diabetes risk extends beyond the effects of generalized adiposity or dyslipidemia, indicating a likely independent pathological pathway. The quartile analysis further strengthened this conclusion, revealing a clear trend of increasing T2DM risk across higher FF-LL categories. The significant P-for-trend in the fully adjusted model solidified the graded nature of this association. This finding is qualitatively consistent with the results reported by Deng et al. (25), yet differs in spatial pattern: whereas Deng observed diffuse hepatic fat elevation across all segments in an obese population, the present study demonstrated predominantly left-lobe fat accumulation in postmenopausal women. This divergence likely reflects heterogeneity in both study population and disease stage; specifically, postmenopausal estrogen decline drives central fat redistribution, and the left lobe, receiving a greater proportion of portal blood enriched with dietary lipids and gut-derived factors, may be more sensitive to early metabolic insult, manifesting initial fat deposition. In contrast, obese individuals with higher overall metabolic load may progress to more diffuse, whole-liver involvement. Our findings suggest that in postmenopausal women with dysglycemia, left-lobe fat fraction may serve as a more sensitive risk biomarker.

ROC curve analysis demonstrated that FF-LL exhibited robust discriminatory ability for differentiating prediabetes from T2DM in postmenopausal women with dysglycemia, with an AUC of 0.816 and an optimal cutoff of 6.3%. Notably, this threshold closely approximates the diagnostic criterion for MASLD (liver fat content ≥5%) (26). The slightly higher cutoff of 6.3% in our study may reflect a more advanced degree of metabolic dysregulation in patients who have already progressed to T2DM compared with the general population. From a clinical perspective, this cutoff provides an objective benchmark for risk stratification: postmenopausal women with dysglycemia and FF-LL ≥ 6.3% may be categorized as high-risk for T2DM and might benefit from more intensive metabolic monitoring and early intervention. These findings underscore the utility of FF-LL as a complementary imaging biomarker for T2DM risk assessment in clinical practice, particularly in postmenopausal women undergoing routine MRI for other indications.

The mediation analysis offers crucial mechanistic insight. The finding that ALT and the AST/ALT ratio mediated approximately 22% and 24.9% of the total effect of FF-LL on T2DM risk is compelling. ALT is a more liver-specific enzyme than AST, and its elevation often reflects hepatocyte injury and inflammation associated with MASLD (27, 28). The AST/ALT ratio is a recognized marker of disease progression in MASLD. This partial mediation implies that hepatic steatosis contributes to T2DM risk not only through mechanisms like increased gluconeogenesis and hepatic insulin resistance but also via pathways involving subclinical hepatic inflammation and cellular injury (29, 30). This aligns with the concept of MASLD as a hepatic manifestation of metabolic syndrome and a driver of its systemic complications, including diabetes (31).

The TyG index is a robust and widely validated surrogate marker for insulin resistance (32). Restricted cubic spline analysis revealed a significant nonlinear association between FF-LL and the TyG index, characterized by a clear threshold effect. Specifically, when the TyG index was below approximately 8.5, FF-LL increased modestly; however, once the TyG index exceeded approximately 8.5, FF-LL exhibited a sharp and sustained rise. This pattern suggests that once insulin resistance surpasses a critical severity threshold, hepatic fat accumulation may enter an accelerated phase. This nonlinear threshold relationship provides important insights into the complex interplay between insulin resistance and hepatic steatosis. From a clinical perspective, it implies that interventions aimed at reducing insulin resistance—particularly in individuals with a TyG index exceeding 8.5—may be especially effective in preventing or slowing hepatic fat accumulation and, consequently, reducing the risk of diabetes. Thus, the TyG index may serve not only as a reliable marker of insulin resistance but also as a practical tool for identifying individuals at risk of accelerated hepatic fat accumulation.

Several limitations of this study must be acknowledged. First, the cross-sectional nature of the analysis precludes definitive causal inferences. While the dose-response gradient and mediation analysis support a plausible causal pathway, longitudinal studies are still needed to confirm that hepatic steatosis precedes and predicts diabetes incidence. Second, the relatively small sample size, particularly within specific quartiles, may limit the statistical power of certain subgroup analyses and affect the generalizability of the findings. Therefore, future prospective studies with larger sample sizes and external validation are needed to confirm our findings. Third, although we adjusted for several key confounders, residual confounding from unmeasured factors (e.g., dietary patterns, physical activity intensity, genetic predisposition) cannot be ruled out. Finally, the study population consisted exclusively of postmenopausal women, limiting the extrapolation of findings to other demographic groups.

In conclusion, FF-LL is an independent T2DM risk factor, with a dose–response relationship partially mediated by liver enzymes (ALT, AST/ALT). At a cutoff of 6.3%, it shows good discriminative ability and a significant nonlinear association with insulin resistance. These findings suggest that FF-LL is a potential biomarker for T2DM risk stratification, though its clinical utility requires external validation in prospective cohorts.

## Data Availability

The raw data supporting the conclusions of this article will be made available by the authors, without undue reservation.
